# Atemwegssicherung bei Neugeborenen und Säuglingen: Handlungsempfehlung gemäß der BJA/ESAIC-Leitlinie

**DOI:** 10.1007/s00101-024-01424-2

**Published:** 2024-07-03

**Authors:** Jost Kaufmann, Dennis Huber, Thomas Engelhardt, Maren Kleine-Brueggeney, Peter Kranke, Thomas Riva, Britta S. von Ungern-Sternberg, Alexander Fuchs

**Affiliations:** 1 Kinderkrankenhaus der Kliniken der Stadt Köln gGmbH, Amsterdamer Str. 59, 50735 Köln, Deutschland; 2 Fakultät für Gesundheit, Universität Witten/Herdecke, Witten, Deutschland; 3 Universitätsklinik für Anästhesiologie und Schmerzmedizin, Inselspital, Universität Bern, Bern, Schweiz; 4 Department of Anesthesiology, Montreal Children’s Hospital, McGill University, Montreal, QC Kanada; 5 Klinik für Kardioanästhesiologie und Intensivmedizin, Deutsches Herzzentrum der Charité (DHZC), Berlin, Deutschland; 6 Charité – Universitätsmedizin Berlin, korporatives Mitglied der Freien Universität Berlin und Humboldt-Universität zu Berlin, Berlin, Deutschland; 7 Klinik und Poliklinik für Anästhesiologie, Intensivmedizin, Notfallmedizin und Schmerztherapie, Universitätsklinikum Würzburg, Würzburg, Deutschland; 8 Department of Anaesthesia and Pain Medicine, Perth Children’s Hospital, Perth, WA Australien; 9 Division of Emergency Medicine, Anaesthesia and Pain Medicine, Medical School, The University of Western Australia, Perth, WA Australien; 10 Institute for Paediatric Perioperative Excellence, The University of Western Australia, Perth, WA Australien; 11 Perioperative Medicine Team, Perioperative Care Program, Telethon Kids Institute, Perth, WA Australien

**Keywords:** Supraglottischer Atwemweg, Bronchoskopie, Muskelrelaxation, Videolaryngoskop, Apnoische Oxygenierung, Supraglottal airway, Bronchoscopy, Muscle relaxation, Video laryngoscope, Apnoic oxygenation

## Abstract

Die Sicherung der Atemwege ermöglicht die Oxygenierung und Ventilation der Lungen und stellt eine potenziell lebensrettende medizinische Maßnahme dar. Insbesondere bei Neugeborenen und Säuglingen kommt es gehäuft zu unerwünschten und kritischen Ereignissen während des Atemwegsmanagements. Die multifaktoriellen Gründe dafür umfassen patientenabhängige, anwenderabhängige, aber auch externe Faktoren. Im Folgenden wird die neu erschienene internationale Leitlinie zur Atemwegssicherung bei Neugeborenen und Säuglingen fokussierend auf die klinische Anwendung zusammengefasst. Während die Originalpublikation der Leitlinie darauf fokussiert, auf Basis der systematisch erfassten Evidenz formale Empfehlungen zu benennen, stellt diese Zusammenfassung v. a. die Praktikabilität ihrer Umsetzung in den Fokus.

**Zusatzmaterial online:**

Die Online-Version dieses Beitrags (10.1007/s00101-024-01424-2) enthält die Zusammenfassung der Leitlinien-Empfehlungen und der Praxistipps.

## Abstract (en)

Securing an airway enables the oxygenation and ventilation of the lungs and is a potentially life-saving medical procedure. Adverse and critical events are common during airway management, particularly in neonates and infants. The multifactorial reasons for this include patient-dependent, user-dependent and also external factors. The recently published joint ESAIC/BJA international guidelines on airway management in neonates and infants are summarized with a focus on the clinical application. The original publication of the guidelines focussed on naming formal recommendations based on systematically documented evidence, whereas this summary focusses particularly on the practicability of their implementation.

## Einleitung

Die Sicherung der Atemwege ist eine Grundvoraussetzung für eine sichere Versorgung von Kindern in der Anästhesie, Intensiv- und Notfallmedizin und ermöglicht die Oxygenierung und Ventilation. Wenn diese nicht gewährleistet ist, kommt es schnell zu Hypoxämie und Bradykardie bis hin zum Herzstillstand. Sedierung und Narkose führen durch einen Verlust des Tonus im Rachen sowie eine Verlagerung des Zungenbeins oft zur Obstruktion der bei Kindern ohnehin schon engen oberen Atemwege. Hinzu kommen besonders bei Früh- und Neugeborenen noch Einschränkungen im Atemantrieb mit einem erhöhten Risiko von reflexbedingten Atempausen oder Spasmen des Larynx, geringere atemmechanische Reserven sowie eine erheblich geringere Apnoetoleranz. Aufgrund der genannten Umstände gehören respiratorische Komplikationen zu den häufigsten Ursachen für perioperative Herzstillstände bei herzgesunden Kindern. Auch bei der Reanimation von Säuglingen und Kleinkindern außerhalb des OP stellt die Beatmung die wichtigste Maßnahme dar. So konnte gezeigt werden, dass – im Gegensatz zum Erwachsenen – bei durch das Telefon angeleiteten Reanimationen und Herzdruckmassagen durch Laien die Überlebensrate um 90 % und die Rate des Überlebens mit gutem neurologischem Ergebnis um mehr als 500 % steigt, wenn die Kinder auch beatmet werden [24]. Der Fokus liegt hierbei immer auf der Aufrechterhaltung der Oxygenierung und der Ventilation, wobei die Maskenbeatmung sowie die Verwendung von supraglottischen Atemwegshilfsmitteln sehr wichtige, weil sehr erfolgreiche Methoden darstellen [44]. Die Gefahr einer bedrohlichen pulmonalen Aspiration ist bei Kindern nicht in gleichem Umfang gegeben wie bei Erwachsenen, und in der Regel profitieren Kinder auch unter Reanimation, bei Schädel-Hirn-Traumen und Polytraumen nicht von einer endotrachealen Intubation [16], solange die supraglottischen Methoden eine suffiziente Ventilation ermöglichen.

Bei Versagen dieser Methoden kann die endotracheale Intubation eine notwendige und lebensrettende Maßnahme sein; zudem ist sie für viele Operationen oder medizinische Interventionen zwingend erforderlich. Insbesondere bei Neugeborenen und Säuglingen kommt es gehäuft zu kritischen Ereignissen während der Intubation [10, 22]. Ursachen für die unerwünschten, bedrohlichen Ereignisse sind patientenabhängige (z. B. Anatomie und Physiologie), anwenderabhängige (z. B. mangelnde Ausbildung und Training) aber auch externe Faktoren (z. B. fehlendes, nichtaltersadäquates Material).

Alle bisherigen Leitlinien zur Atemwegssicherung haben Kinder unter einem Jahr in den Empfehlungen explizit ausgeschlossen, obwohl es gerade in dieser Altersgruppe am häufigsten zu Komplikationen kommt [10]. Daher wurde durch die Europäische Gesellschaft für Anästhesiologie und Intensivmedizin (ESAIC) und die Herausgeber des *British Journal of Anaesthesia* (BJA) eine internationale Expertenkommission gebildet, um evidenzbasierte Handlungsempfehlungen für diese vulnerable Altersgruppe zu erstellen.

Die Kommission hat bedeutsame klinische Fragestellungen zu sieben Themenbereichen der Atemwegssicherung durch endotracheale Intubation identifiziert:

1. Atemwegsbeurteilung zur Erkennung des möglichen schwierigen Atemwegs,
2. optimale Vorbereitung und Planung,
3. Strategien und Techniken zur Intubation beim normalen Atemweg und Abgrenzung vom schwierigen Atemweg,
4. Management des schwierigen Atemwegs,
5. Lagekontrolle des trachealen Tubus,
6. Strategien zur erfolgreichen Extubation
7. Human Factors und personelle Kompetenzen für das Atemwegsmanagement beim Säugling

Gemäß der PICO(„Population, Intervention, Comparison, Outcome“; [29])-Methode wurden sieben klinische Fragestellungen erfasst und zu fünf Elementen der systematischen Literaturrecherche weiterentwickelt (*PubMed, EMBASE, Web of Science* und *Cochrane Central Register of Controlled Trials*; 2011 bis November 2021). Die gefunden Referenzen wurden in formierten Unterarbeitsgruppen von jeweils vier Autoren aus der Expertengruppe unabhängig voneinander auf ihre Relevanz hin überprüft und bei unterschiedlicher Bewertung durch einen weiteren gewichtet. Für weitere Einzelheiten zu den Suchergebnissen, zur systematischen Wertung und Gewichtung der Arbeiten sowie der daraus abgeleiteten wissenschaftlichen Stärke der Empfehlungen (Evidenzgrad) und das Erreichen eines formalen Konsenses verweisen wir auf die Originalpublikation [8, 9].

In diesem Artikel werden die Handlungsempfehlungen der Leitlinie aus klinischer Perspektive zusammenfassend dargestellt. Dabei liegt der Fokus darauf, konkrete und praktikable Empfehlungen für den klinischen Alltag zu benennen. Auf dieser Grundlage kann jede Einrichtung die eigenen Handlungsabläufe überprüfen und überarbeiten.

## Atemwegsbeurteilung zur Erkennung des möglichen schwierigen Atemwegs

### Praxistipp der Autoren.

Wir empfehlen eine gründliche Anamnese und körperliche Untersuchung, um einen schwierigen Atemweg bei Neugeborenen und Säuglingen vorherzusagen.

Eine gezielte Anamnese, eine körperliche Untersuchung sowie die Auswertung aller vorhandenen medizinischen Unterlagen können helfen, mögliche Prädiktoren für einen schwierigen Atemweg frühzeitig zu identifizieren und sich somit adäquat auf die Atemwegssicherung vorzubereiten. Insbesondere das Vorhandensein von Mikro‑, Retro- oder Prognathie, limitierter oder kleiner Mundöffnung, Gesichtsasymmetrien, fixierter Halswirbelsäule, Lippen-Kiefer-Gaumenspalte, Tumoren in Mund, Rachen oder Nacken müssen berücksichtigt werden. Abweichungen von der Norm zu erkennen, erfordert Erfahrung, daher sollten in allen unklaren Fällen Experten zur Beurteilung hinzugezogen werden. Viele der anatomischen Prädiktoren treten vermehrt bei Kindern mit angeborenen Syndromen (z. B. Pierre-Robin‑, Crouzon‑, Treacher-Collins-Syndrom) auf. Studien in diesen Populationen zeigen häufiger eine schwierige Maskenbeatmung durch Obstruktion der oberen Atemwege und seltener eine schwierige Intubation. Insbesondere eine Oxygenierung und Ventilation mittels supraglottischer Atemwegshilfe (SGA) ist zumeist erfolgreich [34, 47]. Viele bekannte Prädiktoren eines schwierigen Atemwegs bei Erwachsenen sind bei Neugeborenen und Säuglingen nicht anwendbar, dies zum einen aufgrund der mangelnden Kooperation, zum anderen aufgrund fehlender Normwerte bei Neugeborenen und Säuglingen.

### Praxistipp der Autoren (nicht in der Original-Leitlinie).

Anamnestisch wertvolle Fragen beim Kind mit Dysmorphie sind:

- Wie sind die Atemgeräusche im Schlaf und beim wachen Kind?
- Gibt es positionsabhängige Atemgeräusche?
- Benötigt das Kind einen speziellen Sauger?
- Muss das Kind altersuntypisch viele Pausen beim Trinken machen?
- Kann es auch mit geschlossenem Mund atmen?

## Optimale Vorbereitung und Planung

### Empfehlung der Leitlinie.

Wir empfehlen, ein angemessenes Maß an Sedierung bzw. eine Allgemeinanästhesie zur Atemwegssicherung. Verabreichen Sie Medikamente zur Blockade der neuromuskulären Überleitung (Muskelrelaxans) vor der trachealen Intubation, wenn eine Spontanatmung nicht erforderlich ist. Dabei sollten die Vor- und Nachteile einer Muskelrelaxation mit der spezifischen Situation von Patient und Team abgewogen werden.

Außer im Rahmen von Wiederbelebungsmaßnahmen (z. B. im Kreißsaal) wurde die endotracheale Intubation bei Neugeborenen und Säuglingen ohne eine adäquate Sedierung und Analgesie in der klinischen Praxis weitgehend verlassen. Argumente für eine Intubation im Wachzustand waren in der Vergangenheit v. a. die Angst vor unerwünschten Wirkungen wie Aspiration, geringer Apnoetoleranz mit rascher Hypoxämie, mangelnde Kenntnis der Pharmakologie und die bedeutenden medizinisch-rechtlichen Auswirkungen in dieser Bevölkerungsgruppe. Eine Intubation beim nicht adäquat medikamentös abgeschirmten Kind ist schmerzhaft und verursacht Stress und ist bei Frühgeborenen mit höheren Raten an Hirnblutungen und Hirninfarkten assoziiert [1].

Zusammenfassend zeigt die Literaturrecherche, dass durch das Verwenden von sedierenden und analgetischen Medikamenten zur Intubation keine Zunahme an unerwünschten Ereignissen zu beobachten ist. Hingegen führt eine adäquate Analgosedierung zu einer Verbesserung der Rate von erfolgreichen Intubationen beim ersten Versuch, einer Verringerung der Anzahl an Versuchen sowie zu einer Reduktion der Inzidenz von Komplikationen [3]. Auch der Einsatz von Muskelrelaxanzien verbessert die Intubationsbedingungen, erhöht die Wahrscheinlichkeit der erfolgreichen Intubationen beim ersten Versuch und verringert die Rate an Komplikationen [13]. Welche medikamentöse Therapie zur neuromuskulären Blockade und zur Analgosedierung bei der Intubation zu priorisieren sind, ist bislang nicht abschließend geklärt. Eine mit altersgruppenspezifisch gewählten, typischen Dosierung von Narkosemedikamenten beginnende und exakte Gabe sowie die individuelle Dosisanpassung unter Wirkungsbeobachtung bis zum Erreichen der angemessenen Analgesie und Sedierung sind notwendig [7].

## Endotracheale Intubation beim normalen Atemweg

### Konventionelle Laryngoskopie oder Videolaryngoskopie

#### Empfehlung der Leitlinie.

Wir empfehlen ein Videolaryngoskop mit einem altersangepassten Standardspatel (Macintosh‑/Miller-Spatel) als erste Wahl für die endotracheale Intubation von Neugeborenen und Säuglingen.

Durch die Verwendung eines Videolaryngoskops kann die Rate an erfolgreichen Intubationen im ersten Versuch bei Säuglingen erhöht werden [15]. Auch in einem Vergleich einer Videolaryngoskopie mit der direkten Laryngoskopie bei Neugeborenen und Säuglingen, bei dem beiden Gruppen eine apnoische Sauerstoffzufuhr erfolgte, war die Rate an erfolgreichen endotrachealen Intubationen beim ersten Versuch ohne Entsättigung signifikant höher [37]. Ein weiterer, bedeutsamer Vorteil besteht durch die Verwendung von Videolaryngoskopen darin, dass Lernende besser angeleitet werden können, was die Rate an erfolgreichen Intubationen beim ersten Versuch im Vergleich zur konventionellen Laryngoskopie je nach Konstellation steigert [35] und bis hin zu verdoppeln bis verdreifachen kann [48]. Um auch mit konventionellen Laryngoskopen zurechtzukommen, sollten die Lernenden nicht ausschließlich die Videofunktion benutzen, sondern auch den direkten Blick verwenden.

#### Praxistipp der Autoren.

Ein Videolaryngoskop sollte bei der Ausbildung verwendet werden, und zwar mit einem „dualen Ansatz“: direkte Laryngoskopie für den Auszubildenden und Videolaryngoskopie für den Anleitenden.

Bezüglich der Form des Spatels (Miller- oder Macintosh-Spatel) kann weder bei der konventionellen Laryngoskopie noch mit Videolaryngoskopen eine grundsätzliche Über- oder Unterlegenheit festgestellt werden, jedoch kann bei schlechter Visualisierung der Glottis ein Wechsel des Spatels die Sicht verbessern [46].

### Apnoische Oxygenierung während der endotrachealen Intubation

#### Empfehlung der Leitlinie.

Wir empfehlen die apnoeische Oxygenierung während der endotrachealen Intubation bei Neugeborenen.

Bei der apnoeischen Oxygenierung wird Sauerstoff in den Atemweg verabreicht, ohne dass eine Ventilation stattfindet. Dazu sind verschiedene Methoden mit unterschiedlichen Flussraten des Sauerstoffzustroms sowie unterschiedlichen Applikationswegen möglich. Nach aktuellem Kenntnisstand besteht kein bedeutsamer Unterschied, wie und mit welchen Flussraten der Sauerstoff während der Laryngoskopie zur endotrachealen Intubation verabreicht wurde [8, 9]. Grundsätzlich erhöht eine apnoische Oxygenierung nach Beendigung der Maskenbeatmung während der endotrachealen Intubation die Rate an erfolgreichen Intubationen beim ersten Versuch, verlängert die sichere Apnoezeit und senkt die Inzidenz von Hypoxämien [14, 38]. Relativ einfach praktikabel ist es, den Sauerstofffluss z. B. mit 5 l/min über eine Nasenkanüle zu verabreichen.

#### Praxistipp der Autoren.

Verwenden Sie auch bei Säuglingen die apnoeische Oxygenierung, angepasst an das patientenspezifische Risiko für eine Hypoxämie sowie die Erfahrung des ausführenden Teams.

### Endotrachealtubus mit oder ohne Cuff

#### Empfehlung der Leitlinie.

Wir empfehlen die Verwendung eines Tubus mit oder ohne Cuff jeweils so, wie es in der Gesamtkonstellation am passendsten erscheint. Bei Kindern über 3 kg können sowohl gecuffte als auch ungecuffte Tuben sicher angewendet werden.

Bei der Verwendung eines Endotrachealtubus mit Cuff ist es seltener notwendig, aufgrund einer Undichtigkeit des Atemweges den Tubus zu wechseln. Dies ist insbesondere in Notfallsituationen und bei unerfahrenen Anwendern von Vorteil. Um eine Schädigung des Larynx und einen Postextubationstridor zu verhindern, müssen die Größenempfehlungen der Hersteller beachtet und bei gecufften Tuben auf die Grenzen des Cuff-Drucks unter Beachtung der Herstellerangaben geachtet werden (Cuff-Druck nie mehr als 20–30 cm H_2_O) [6, 49]. Je nach Art und Hersteller des Tubus variieren die Außendurchmesser teilweise erheblich bei gleicher nomineller Tubusgröße (Innendurchmesser), was ebenso zu beachten ist.

### Nasale oder orale endotracheale Intubation

Manchmal wird bei Neugeborenen, insbesondere wenn eine verlängerte Intubation antizipiert wird, die nasale Intubation bevorzugt, hingegen bei Säuglingen für kurzfristige Beatmung eher die orale [43]. Evidenz liegt hierfür nicht vor, auch wenn es nachvollziehbare Argumente gibt. Gerade für wenig Erfahrene und in einer Notfallsituation ist die orale Passage des Tubus oft einfacher. Ein nasal platzierter Tubus ist jedoch besser zu fixieren und vereinfacht die Mundpflege und das Sedierungs-Weaning auf der Intensivstation.

#### Praxistipp der Autoren.

Bei der nasalen Intubation sollten ein erhöhtes Blutungsrisiko berücksichtigt und die Gabe topischer Vasokonstriktoren vorab erwogen werden.

### Supraglottische Atemwegshilfe als Alternative zur Intubation

#### Empfehlung der Leitlinie.

Eine supraglottische Atemwegshilfe ist ein effizientes Hilfsmittel zu Oxygenierung und -ventilation.

Die supraglottische Atemwegshilfe (SGA) in Form einer Larynxmaske ermöglicht zumeist eine Beatmung von Neugeborenen und kleinen Säuglingen. Als alternative SGA kann auch ein nasopharyngeal positionierter Endotrachealtubus zur Beatmung verwendet werden („Pharynxtubus“), dann müssen das gegenüberliegende Nasenloch und der Mund zugehalten oder mir der Handfläche des Anwenders abgedichtet werden. Die supraglottische Atemwegshilfe kann im Vergleich zum endotrachealen Tubus schneller und häufiger beim ersten Versuch richtig platziert werden. Bei einer kardiopulmonalen Reanimation sind eine Maskenbeatmung oder ein supraglottischer Atemweg bezüglich der Überlebensrate oder dem neurologischen Outcome der trachealen Intubation nicht unterlegen [16]. Auch im elektiven operativen Setting kann durch die Verwendung einer SGA die Inzidenz von leichten oder schwerwiegenden respiratorischen Komplikationen erheblich gesenkt werden [11].

## Management des schwierigen Atemwegs

Die Expertengruppe der Leitlinie schlägt vor, eine schwierige Intubation als zwei fehlgeschlagene endotracheale Intubationsversuche zu definieren. Dies würde die Vergleichbarkeit von Studien und die Bewertung der Wirksamkeit von Maßnahmen verbessern.

### Empfehlung der Leitlinie.

Halten Sie für das Management antizipierter schwieriger Atemwege mindestens ein Videolaryngoskop, ein flexibles Intubationsbronchoskop und ein rigides oder semirigides Intubationsbronchoskop in altersentsprechenden Größen bereit (zusätzlich zu den routinemäßig verwendeten Geräten und Hilfsmitteln wie Gesichtsmasken, Guedel-Tubus und supraglottischen Atemwegshilfsmitteln).

Es gibt keine Patentlösung („one solution fits all“) für das Management schwieriger Atemwege bei Neugeborenen und Säuglingen. In jedem medizinischen Versorgungsbereich, in dem Säuglinge beatmet werden müssen, müssen alle gängigen Geräte für die Beutel-Masken-Beatmung, die Öffnung der oberen Atemwege (z. B. durch Guedel- oder Wendl-Tuben), supraglottische Atemwegshilfsmittel, Absaugkatheter, Laryngoskope und Trachealtuben in geeigneten Größen zur Verfügung stehen.

Mehrere Intubationsversuche mit derselben Technik verursachen eine höhere Wahrscheinlichkeit von Komplikationen (Hypoxämie, Bradykardien, Hypo- oder Hypertensionen) und können Schwellung und Blutung der Atemwege verursachen.

### Empfehlung der Leitlinie.

Limitieren Sie die Anzahl der (identischen) Intubationsversuche, indem Sie nach jedem Versuch die Umstände überprüfen und verbessern sowie zu einer anderen Technik oder/und einem anderen Anwender wechseln.

Aus wiederholten gleichen Versuchen resultieren geringere Erfolgschance bei nachfolgenden Intubationsversuchen mit derselben oder einer anderen Technik. Die Rate von erfolgreichen Intubationen beim ersten Versuch ist größer, je mehr Erfahrung die ausführende Person hat [19, 27].

### Praxistipp der Autoren.

In Fällen, in denen eine Laryngoskop mit einer Standardspatelform versagt und der Atemweg erwartungsgemäß schwierig ist (z. B. Retrognathie, eingeschränkte Mundöffnung oder Beweglichkeit der Halswirbelsäule), sollte als nächster Schritt zu einer alternativen fortgeschrittenen Technik übergegangen werden, die die Verwendung von hyperangulierten Spateln mit einem Stilett und eine flexible oder starre Bronchoskopie allein oder in Kombination mit einem Videolaryngoskop oder eine flexible Bronchoskopie über einen supraglottischen Atemweg umfasst.

Da die Ursache der Atemwegsschwierigkeiten individuell verschieden ist, können die Atemwegseinschränkungen des einzelnen Patienten zwangsläufig eine der oben genannten Instrumente für den Atemweg zwingend erfordern. Beispielsweise kann es bei erheblichen Engstellen im subglottischen Raum (z. B. durch Zysten) oder Schwellungen im supraglottischen Raum (z. B. durch Ödeme oder Infektionen) schwierig oder unmöglich sein, mit einem flexiblen Bronchoskop gegen einen Widerstand zu intubieren, und nur mit (semi-)rigiden Optiken möglich sein.

### Empfehlung der Leitlinie.

Verwenden Sie eine rigide oder semirigide Optik, wenn ein Raum bei der Passage des Tubus (durch eine Stenose, Schwellung oder Kompression) verengt oder verlegt ist.

Zudem kann eine endotracheale Intubation mit rigiden Optiken oft schneller durchgeführt [21] und auch schneller erlernt werden, als dies mit flexiblen Bronchoskopen der Fall ist [42]. Grundsätzlich können aber die meisten Atemwegsprobleme mit einer flexiblen Optik gelöst werden, daher ist dies sicher das bedeutsamste erweiterte Instrument zur endotrachealen Intubation. Zu beachten ist, dass flexible Bronchoskope nur an der Spitze steuerbar und im Verlauf rein materialabhängig biegsam sind; dies ist durch den Instrumentierenden nicht zu beeinflussen. Dies kann die Navigation mit der flexiblen Optik gegen einen Widerstand unmöglich machen. Zusätzlich kann diese Flexibilität dazu führen, dass, wenn der Tubus über das liegende Endoskop vorgebracht wird und sich an der Supraglottis verhakt (typischerweise an den Aryknorpeln), bei weiterem Vorschieben des Tubus eine Kurve im Bronchoskop oberhalb des Larynx entsteht und die Optik aus der Trachea herausgezogen wird. Dies ist besonders bei einem großen Kalibersprung zwischen der Optik und dem Tubus häufig.

Die flexible Intubation durch eine supraglottische Atemwegshilfe findet im klinischen Alltag eine weite Verbreitung und in der Literatur zu Recht viel Anerkennung. Je kleiner Kinder sind, desto schwieriger ist aber die Navigation einer mit einem Tubus bestückten flexiblen Optik durch eine supraglottische Atemwegshilfe. Durch die kleinen Räume innerhalb der Hilfsmittel und die Reibung der Materialien aneinander kann die endotracheale Platzierung des Tubus erheblich erschwert oder sogar unmöglich sein. Vorab muss zwingend das vorhandene Equipment auf Kompatibilität überprüft werden – welche Larynxmaske passt mit welchem Tubus und welcher Fiberoptik?

Zudem muss nach erfolgreicher endotrachealer Intubation die über dem Tubus liegende supraglottische Atemwegshilfe entfernt werden. Dafür sind verschiedene Techniken beschrieben, mit denen der Tubus in Position gehalten wird, zum Beispiel mit einem zweiten, zuvor zusätzlich auf das Bronchoskop umgedreht aufgezogenen Tubus. Durch Herabdrücken dieses zweiten Tubus soll der erste in Position gehalten werden. Gleiches kann auch durch Sichern des endotrachealen Tubus mit einem Laryngoskop und einer Magill-Zange geschehen, sobald Platz im Mundraum entsteht. Egal, welche Methode verwendet wird, die Entfernung der supraglottischen Atemwegshilfe aus dem Atemweg und der Erhalt des in ihr liegenden endotrachealen Tubus sind mit Aufwand und der grundsätzlichen Gefahr einer Tubusdislokation verbunden.

### Praxistipp der Autoren.

Nach vier Versuchen sollte erwogen werden, die Intubation abzubrechen und den Patienten aufzuwecken, wenn dies möglich ist.

Der Gebrauch von Laryngoskopen oder Videolaryngoskopen mit einem hyperangulierten Spatel ermöglicht häufiger eine bessere Sicht auf die Glottis, wenn normal konfigurierte Spatel scheitern.

### Empfehlung der Leitlinie.

Wir empfehlen die Verwendung eines Führungsdrahts zu Formung und Stabilisierung des Tubus bei der Verwendung eines Videolaryngoskops mit hyperanguliertem Spatel oder bei anteriorem Larynx.

Bei der Laryngoskopie mit einem hyperangulierten Spatel befindet sich der Larynx trotz guter Visualisierung vor der oralen Achse und es gibt keinen enoralen Schnittpunkt der oralen und trachealen Achse, wie bei der Verwendung von regulären Spateln und guter Sicht. Daher ist die Passage des Tubus in die Stimmbandebene grundsätzlich erschwert und erfordert eine stärkere Kurve und somit die Verwendung eines Führungsdrahtes oder Mandrins zur Vorformung des Tubus (Anpassung an die Krümmung des Spatels). Die Passage des Endotrachealtubus kann regelhaft trotz einer guten Visualisierung dennoch unmöglich sein. Aus gleichem Grund führt insbesondere bei Kindern < 5 kg die Verwendung eines Videolaryngoskops mit einem konventionell geformten Spatel zu einer höheren Erfolgsquote beim ersten Versuch [36].

### Empfehlung der Leitlinie.

Verwenden Sie bei stark eingeschränkter Mundöffnung, die eine enorale Instrumentierung verhindert, flexible Fiberoptiken zur Intubation.

### Praxistipp der Autoren.

Eine durchgehende Beatmung ist während der fiberoptischen Intubation möglich, indem spezielle Endoskopie-Masken (z. B. Frei-Maske) oder Winkel-Konnektoren (z. B. Swivel-Konnektor, Mainzer Adapter) verwendet werden [18] oder ein „Pharynxtubus“ parallel verwendet wird.

Das bei Erwachsenen im Falle einer „Cannot-ventilate-cannot-intubate“ Situation empfehlenswerte Verfahren der perkutanen Notfallkoniotomie (in der Membrana cricothyroidea) ist bei Neugeborenen und Säuglingen ungeeignet [2]. Aufgrund des hoch sitzenden Kehlkopfes nah am Kinn ist ein sehr steiler Punktionswinkel notwendig und eine Kompression des Tracheallumens hierbei unvermeidbar. Weil die Atemwege bei Säuglingen hier zudem sehr eng sind und die Membrana cricothyroidea sehr klein ist, ist diese Technik nicht erfolgversprechend. Die Trachea liegt hingegen weiter abwärts, hat ein größeres Lumen und ist in der Regel gut zu ertasten.

### Praxistipp der Autoren.

Führen Sie (statt wie bei Erwachsenen eine Koniotomie) eine chirurgische Tracheotomie durch, wenn nach einer Narkoseeinleitung die Intubation nicht möglich, eine Beatmung mit einer Gesichtsmaske oder einem supraglottischen Atemwegshilfsmittel nicht funktioniert und eine Rückkehr zur Spontanatmung nicht möglich ist.

Von all den verschiedenen bisher beschriebenen Techniken der chirurgischen Tracheotomie kann keine als den anderen überlegen gewertet werden.

### Praxistipp der Autoren.

Wenn Fachwissen und Kompetenz vorhanden sind, kann die extrakorporale Membranoxygenierung (ECMO) als Rettungsmaßnahme in Betracht gezogen werden.

Die Entscheidung und der Zeitpunkt für die Etablierung einer ECMO erfolgen individuell und müssen den einzelnen Anwendern überlassen werden. Da die Etablierung einer ECMO auch in hochspezialisierten Zentren Zeit braucht, ist eine frühzeitige Alarmierung des ECMO-Teams zu erwägen.

## Lagekontrolle des trachealen Tubus

### Empfehlung der Leitlinie.

Überprüfen Sie eine erfolgreiche Intubation sofort anhand der klinischen Beurteilung der Beatmung und einer kontinuierlichen endtidalen CO_2_-Detektion mit steigender Wellenform.

Nach schwierigen Intubationen oder bei komplexen Patienten kann zusätzlich die optimale Position der Tubusspitze in der Trachea durch eine erneute Videolaryngoskopie mit Darstellung der Einführtiefe des Tubus, eine Thoraxröntgenuntersuchung, eine fiberoptische Darstellung der Tubusspitze vor der Carina oder eine Ultraschalluntersuchung in Betracht gezogen werden [32, 45].

## Strategien zur erfolgreichen Extubation

Die Extubation ist ein elektiver und planbarer Moment, für den dementsprechend eine vollständige Vorbereitung stattfinden sollte.

### Praxistipp der Autoren.

Bei jeder Extubation muss die Ausrüstung für eine sofortige Reintubation vorhanden sein, wobei die spezifischen Besonderheiten des Kindes, der vorherigen Atemwegssicherung und die Ursachen einer vorherigen schwierigen Intubation bei dieser Vorbereitung berücksichtigt werden müssen.

Aus der Wertung dieser Umstände ergeben sich das Equipment, welches zur potenziellen Reintubation bereitgestellt werden muss, sowie die Abschätzung, ob eine medikamentöse Vorbehandlung vor der Extubation verabreicht wird. In Studien wurde zusammenfassend gezeigt, dass durch das Vernebeln von Epinephrin zwar nicht ein Postextubationsstridor, aber die Wahrscheinlichkeit einer Reintubation verhindert werden kann [4]. Die Gabe von Kortikosteroiden kann zur Vermeidung eines Postextubationsstridors beitragen [23].

### Praxistipp der Autoren.

Verwenden Sie Kortikosteroide und/oder vernebeltes Epinephrin zur Vorbeugung und zur Behandlung von Stridor nach der Extubation, wenn die Atemwege erheblich manipuliert wurden.

Für die Beurteilung der Extubationsbereitschaft sind v. a. klinische Faktoren ausschlaggebend (Grimassieren, Augen öffnen, fokussierender Blick, gezielte Bewegungen und ein Tidalvolumen > 5 ml/kgKG), wobei die Sinnhaftigkeit dieses klinisch erfolgreichen Vorgehens auch durch Studien bestätigt wurde [41]. Bei der Verwendung von Muskelrelaxanzien sollten immer auch deren Wirkung und deren Wirkungsende durch eine Messung der neuromuskulären Übertragung überwacht werden. Die modernere Methode der Messung durch eine Elektromyographie ist der akzelerometrischen Messung bei Säuglingen überlegen.

Nach der Extubation kann es notwendig werden, die Atmung der Kinder vorrübergehend zu unterstützen, um eine Reintubation zu verhindern. Durch den Gebrauch von nasalem High-Flow-Sauerstoff (HFNO), kontinuierlichem positivem Atemwegsdruck (CPAP) sowie einer nasalen nichtinvasiven Beatmung (nNIV) kann diese Wahrscheinlichkeit signifikant gesenkt werden [28].

### Empfehlung der Leitlinie.

Verwenden Sie nichtinvasive Unterstützung der Atmung, falls dies nach der Extubation notwendig ist (nasale High-Flow-Oxygenierung, kontinuierliche positive Atemwegsbeatmung oder nasale intermittierende Überdruckbeatmung).

### Schlafende oder wache Extubation

Ob die Extubation oder das Entfernen eines supraglottischen Atemwegshilfsmittels (SGA) im wachen oder im schlafenden Zustand erfolgen soll, kann aus der Literatur nicht eindeutig beantwortet werden. Dies liegt am Fehlen einer klaren Definition zwischen schlafender und wacher Extubation sowie an den Studien, die Populationen von Kindern aller Altersstufen untersuchen [31]. Die schlafende Extubation war in einer Metaanalyse mit einer geringeren Inzidenz von Atemwegskomplikationen und Entsättigungen assoziiert [25]. Weil es aber häufiger zu einer Verlegung der oberen Atemwege bei früher Extubation kommt, ist dieses Vorgehen nur in einem Setting, in dem diese Obstruktion durch erfahrenes Personal sicher beherrscht werden kann, empfehlenswert.

#### Praxistipp der Autoren.

Wenn die Maskenbeatmung und die Intubation problemlos waren sowie keine klinischen erhöhten Risiken für Atemwegskomplikationen (Infobox) vorliegen, sind eine altersgerechte Atemfrequenz mit Tidalvolumina von mindestens 5 ml/kgKG Kriterien für die Extubation.

Für die Entscheidung zu einer frühen Entfernung in Narkose oder später und entsprechend wach sollten individuelle Aspekte für das Risiko von Atemwegskomplikationen herangezogen werden.

#### Infobox Risiken für Atemwegskomplikationen (die für eine späte, wache Extubation sprechen)

- Bei der Versorgung von Kindern unerfahrene Ausführende, Verfügbarkeit von Ressourcen und Unterstützung
- Atemwegskomplikationen, eine schwierige oder traumatische Intubation
- Individuell hohes Risiko für Atemwegskomplikationen (manifeste Atemwegsinfektion, Asthma, Anomalien der Atemwege, verminderte pulmonale Reserven, thorakoabdominelle Asynchronität, altersentsprechend niedrige Atemfrequenz, verminderter muskulärer [Rachen-]Tonus, hoher Sauerstoffbedarf)
- Bedeutsame Komorbiditäten (Herzerkrankungen, Stoffwechselerkrankungen, Sepsis, Frühgeburtlichkeit)
- Eingriffe in der Nähe der Atemwege (z. B. Kiefer‑, Gesichts‑, Hals-Nasen-Ohren‑, Neuro- oder plastische Chirurgie)
- Hohes Risiko für Aspirationen (Notfalleingriff mit nichtnüchternem Kind, relevanter gastroösophagealer Reflux, Achalasie)
- Keine vollständige Erholung von einer neuromuskulären Blockade

## Human Factors und personelle Kompetenzen für den pädiatrischen Atemweg

Vergleichbar mit anderen komplexen Arbeitsbereichen und Systemen mit häufigen Mensch-Maschinen-Interaktionen treten unerwünschte Komplikationen in der Anästhesie häufig als ein Ergebnis von unvorhersehbaren Kombinationen aus menschlichem und organisatorischem Versagen auf. Verschiedene menschliche Faktoren sind in der Anästhesie bedeutsam für die Patientensicherheit. Darunter fallen Teamarbeit, Kommunikation, Situationsbewusstsein, eine positive Fehlerkultur sowie Hierarchien, die bidirektionale Kommunikation und Kritik zulassen [20]. Kognitive Prozesse bei der Entscheidungsfindung wie ein fehlendes Risikobewusstsein, persönliche Selbstüberschätzung, Fixierungsfehler oder auch Verlustangst spielen eine entscheidende Rolle in dem schnell veränderlichen und komplexen Arbeitsumfeld der Anästhesie [40].

### Praxistipp der Autoren.

Eine gute Zusammenarbeit im Team, zielgerichtete Kommunikation sowie eine Atmosphäre mit einer gesunden Fehlerkultur können Patientenschäden reduzieren.

Untersuchungen der pädiatrischen Anästhesie zeigen, dass über die Hälfte aller kritischen Zwischenfällen auf menschliche Faktoren zurückzuführen sind und über die Hälfte der kritischen Zwischenfälle respiratorische Ereignisse sind [26]. Kognitive Hilfen wie Algorithmen und Checklisten können zu einer strukturierten Lösungsfindung in kritischen Situationen beitragen. Durch strukturelle Optimierung des Systems, Erhöhung der Erfahrung des Teams [17] und simulationsgestütztes Team-Training [50], welches auch nichttechnische Fertigkeiten, wie Resilienz und Stressbewältigung, inkludiert, kann eine Verbesserung der Patientensicherheit und -versorgung in der klinischen Praxis wirksam umgesetzt werden.

### Praxistipp der Autoren.

Das Atemwegsmanagement bei Neugeborenen und Säuglingen erfordert eine Reihe spezifischer Fähigkeiten und ein strukturiertes Training. Es ist ratsam, die Fähigkeiten mithilfe eines Simulationstrainings zur neonatalen und Säuglingsintubation zu implementieren und aufrechtzuerhalten.

### Maskenbeatmung (nicht in der Original-Leitlinie)

Die Maskenbeatmung stellt die bedeutendste Maßnahme bei der Atemwegssicherung von Neugeborenen und Säuglingen dar. Sie ist die häufigste angewendete Technik in der elektiven Atemwegssicherung, und insbesondere in Notfallsituationen die erste und am schnellsten verfügbare Möglichkeit, um die Oxygenierung und Ventilation wiederherzustellen oder aufrechtzuerhalten [30, 39]. Die Schulung der Maskenbeatmung sollte sich darauf fokussieren, eine suffiziente Technik anhand der direkten Patientenbeobachtung zu erkennen und Maßnahmen zu kennen, diese gegebenenfalls zu optimieren. Erfahrene Personen können die Maskenbeatmung auf Grundlage von visueller Patientenbeobachtung effektiver anpassen, selbst mit limitiertem technischem Equipment [33], sodass diese Fähigkeit bei der Ausbildung und im Training fokussiert werden muss. Dies erscheint umso wichtiger, weil bei der schwierigen Maskenbeatmung ein erhöhtes Risiko für Hypoxämien besteht [5, 6]. Wenn es schwierig ist, den Patienten mittels Maskenbeatmung mit Sauerstoff zu versorgen, ist es unerlässlich, anatomische und funktionelle Atemwegsobstruktionen auszuschließen (Tab. 1; [12]).

**Tab. 1:** Ursachen für eine schwierige Maskenbeatmung und passende Maßnahmen

Ursache	Maßnahme
*Anatomische/mechanische Ursachen*	
Nichtadäquate Kopfposition	Neupositionierung
Große Adenoide/Tonsillen/Adipositas	Oropharyngealer^a^/nasopharyngealer^b^ Tubus
	Zweihand‑/Zweipersonentechnik^c^
Blut, Fremdkörper, Sekrete	Absaugen, Entfernen
Alveolarkollaps („closing capacity“)	Alveolare Rekrutierungsmanöver^d^
Magenüberblähung/Distension	Dekompression durch Magensonde
*Funktionelle Atemwegsobstruktionen*	
Unzureichende Narkosetiefe	Vertiefung der Narkose
Laryngospasmus	Propofol, Muskelrelaxanzien
Opioidinduzierte Muskelrigidität und/oder Stimmbandverschluss	Muskelrelaxanzien
Bronchospasmus	Epinephrin, Bronchodilatatoren (Sevofluran)

^a^Guedel-Tubus

^b^Wendl-Tubus

^c^Esmarch-Handgriff

^d^Z. B. durch Atemhübe mit verlängerter Inspirationszeit und erhöhtem Inspirationsdruck

## Fazit für die Praxis

Die aktuelle Leitlinie für das Atemwegsmanagement von Neugeborenen und Säuglingen bietet Handlungsempfehlungen auf der Basis einer systematischen Analyse der vorhandenen Evidenz. Dadurch wurden sicherheitsrelevante Empfehlungen mit einem starken Einfluss auf die Patientensicherheit identifiziert. Somit bieten die Leitlinien eine wertvolle Grundlage für eine Bewertung und Überarbeitung der eigenen instrumentellen, personellen und strukturellen Gegebenheiten, die von den anwendenden Personen anhand ihrer individuellen Strukturen konkret umgesetzt müssen. Die vorliegende praxisnahe Darstellung enthält zusätzliche klinische Tipps und Anregungen, welche die lokale Adaptation erleichtern können. Neben der Auswahl der instrumentellen Ausrüstung und Umsetzung der strukturellen Maßnahmen kommt der Beachtung der menschlichen Faktoren und deren Verbesserung eine zentrale Bedeutung zu. Durch Etablierung einer hohen Sicherheitskultur und Verfestigung dieser durch regelmäßiges Team-Training kann die Sicherheit beim Atemwegsmanagement von Neugeborenen und Säuglingen in jeder einzelnen Einrichtung optimiert werden.

### Supplementary Information

Zusammenfassung der Leitlinien-Empfehlungen und der Praxistipps
