# Supplementary material for: Airway management in neonates and infants: Recommendations according to the ESAIC/BJA guidelines
Source: Anaesthesiologie. 2024 Jul 3;73(7):473–81. [Article in German] doi: 10.1007/s00101-024-01424-2 (PMC11222175; doi:10.1007/s00101-024-01424-2)
Supplement: Supplementary file 1 — Zusammenfassung der Leitlinien-Empfehlungen und der Praxistipps [file 101_2024_1424_MOESM1_ESM.pdf]

Zusatzmaterial zum Beitrag **„Atemwegssicherung bei Neugeborenen und Säuglingen. Handlungsempfehlung gemäß der BJA/ESAIC-Leitlinie“** von Kaufmann J, Huber D, Engelhardt T et al. (2024) in *Die Anaesthesiologie*

Beitrag und Zusatzmaterial stehen Ihnen auf www.springermedizin.de zur Verfügung. Bitte geben Sie dort den Beitragstitel in die Suche ein.

Zusammenfassung der Leitlinien-Empfehlungen und der Praxis-Tipps

**Atemwegsbeurteilung zur Erkennung des möglichen schwierigen Atemwegs**	**Empfehlung der Leitlinie:**<br>• Wir empfehlen eine gründliche Anamnese und körperliche Untersuchung, um einen schwierigen Atemweg bei Neugeborenen und Säuglingen vorherzusagen.<br>**Praxistipp der Autoren:**<br>• Anamnestisch wertvolle Fragen beim Kind mit Dysmorphie sind:<br>• Wie sind die Atemgeräusche im Schlaf und beim wachen Kind?<br>• Gibt es positionsabhängige Atemgeräusche?<br>• Benötigt das Kind einen speziellen Sauger?<br>• Muss das Kind altersuntypisch viele Pausen beim Trinken machen?<br>• Kann es auch mit geschlossenem Mund atmen?
**Optimale Vorbereitung und Planung**	**Empfehlung der Leitlinie:**<br>• Wir empfehlen, ein angemessenes Maß an Sedierung bzw. eine Allgemeinanästhesie zur Atemwegssicherung. Verabreichen Sie Medikamente zur Blockade der neuromuskulären Überleitung (Muskelrelaxans) vor der trachealen Intubation, wenn eine Spontanatmung nicht erforderlich ist. Dabei sollten die Vor- und Nachteile einer Muskelrelaxation abgewogen werden mit der spezifischen Situation von Patient und Team.
**Strategien und Techniken zur Intubation beim normalen Atemweg und Abgrenzung vom schwierigen Atemweg**	**Konventionelle Laryngoskopie oder Videolaryngoskopie**<br>**Empfehlung der Leitlinie:**<br>• Wir empfehlen ein Videolaryngoskop mit einem altersangepassten Standardspatel (Macintosh/Miller) als erste Wahl für die endotracheale Intubation von Neugeborenen und Säuglingen.<br>**Praxistipp der Autoren:**<br>• Ein Videolaryngoskop sollte bei der Ausbildung verwendet werden, und zwar mit einem "dualen Ansatz": Direkte Laryngoskopie für den Auszubildenden und Videolaryngoskopie für den Anleitenden.<br><br>**Apnoische Oxygenierung während der endotrachealen Intubation**<br>**Empfehlung der Leitlinie:**<br>• Wir empfehlen die apnoeische Oxygenierung während der endotrachealen Intubation bei Neugeborenen.

**Strategien und Techniken zur Intubation beim normalen Atemweg und Abgrenzung vom schwierigen Atemweg**	**Praxistipp der Autoren:**<br>• Verwenden Sie auch bei Säuglingen die apnoeische Oxygenierung angepasst an das patientenspezifische Risiko für eine Hypoxämie sowie die Erfahrung des ausführenden Teams.<br><br>**Endotrachealtubus mit oder ohne Cuff**<br>**Empfehlung der Leitlinie:**<br>• Wir empfehlen die Verwendung eines Tubus mit oder ohne Cuff jeweils so, wie es in der Gesamtkonstellation am passendsten erscheint. Bei Kindern über 3 kg können sowohl gecuffte als auch ungecuffte Tuben sicher angewendet werden.<br><br>**Nasale oder orale endotracheale Intubation**<br>**Praxistipp der Autoren:**<br>• Bei der nasalen Intubation sollte ein erhöhtes Blutungsrisiko berücksichtigt und die Gabe topischer Vasokonstriktoren vorab erwogen werden.<br><br>**Supraglottische Atemwegshilfe als Alternative zur Intubation**<br>**Empfehlung der Leitlinie:**<br>• Eine supraglottische Atemwegshilfe ist ein effizientes Hilfsmittel zur Oxygenierung und -ventilation.
**Management des schwierigen Atemwegs**	**Empfehlung der Leitlinie:**<br>• Halten Sie für das Management antizipierter schwieriger Atemwege mindestens ein Videolaryngoskop, ein flexibles Intubations-Bronchoskop und ein rigides oder semi-rigides Intubations-Bronchoskop in altersentsprechenden Größen bereit (zusätzlich zu den routinemäßig verwendeten Geräten und Hilfsmitteln wie Gesichtsmasken, Guedel und supraglottischen Atemwegshilfsmitteln).<br>• Limitieren Sie die Anzahl der (identischen) Intubationsversuche, indem Sie nach jedem Versuch die Umstände überprüfen und verbessern, sowie zu einer anderen Technik oder/und einen anderen Anwender wechseln.<br>• Verwenden Sie eine rigide oder semi-rigide Optik, wenn ein Raum bei der Passage des Tubus (durch eine Stenose, Schwellung oder Kompression) verengt oder verlegt ist.<br>• Wir empfehlen die Verwendung eines Führungsdrahts zur Formung und Stabilisierung des Tubus bei der Verwendung eines Videolaryngoskops mit hyperanguliertem Spatel oder bei anteriorem Larynx.<br>• Verwenden Sie bei stark eingeschränkter Mundöffnung, die eine enorale Instrumentierung verhindert, flexible Fiberoptiken zur Intubation.<br><br>**Praxistipp der Autoren:**<br>• In Fällen, in denen eine Laryngoskop mit einer Standard-Spatelform versagt und der Atemweg erwartungsgemäß schwierig ist (z.B. Retrognathie, eingeschränkte Mundöffnung oder Beweglichkeit der Halswirbelsäule), sollte als nächster Schritt zu einer alternativen fortgeschrittenen Technik übergegangen werden, die die Verwendung von hyperangulierten Spateln mit einem Stilett und eine flexible oder starre Bronchoskopie allein oder in Kombination mit einem Videolaryngoskop oder eine flexible Bronchoskopie über einen supraglottischen Atemweg umfasst.<br>• Nach vier Versuchen sollte erwogen werden, die Intubation abzubrechen und den Patienten aufzuwecken, wenn dies möglich ist.

**Management des schwierigen Atemwegs**	• Eine durchgehende Beatmung ist während der fiberoptischen Intubation möglich, indem spezielle Endoskopie-Masken (z.B. Frei-Maske) oder Winkel-Konnektoren (z.B. Swivel-Konnektor, Mainzer Adapter) verwendet werden [18] oder ein nasopharyngeal positionierter Endotrachealtubus („Pharynxtubus“) parallel verwendet wird.<br>• Führen Sie (statt wie bei Erwachsenen eine Koniotomie) eine chirurgische Tracheotomie durch, wenn nach einer Narkoseeinleitung die Intubation nicht möglich, eine Beatmung mit einer Gesichtsmaske oder einem supraglottischen Atemwegshilfsmittel nicht funktioniert und eine Rückkehr zur Spontanatmung nicht möglich ist.<br>• Wenn Fachwissen und Kompetenz vorhanden sind, kann die extrakorporale Membranoxygenierung (ECMO) als Rettungsmaßnahme in Betracht gezogen werden.
**Lagekontrolle des trachealen Tubus**	**Empfehlung der Leitlinie:**<br>• Überprüfen Sie eine erfolgreiche Intubation sofort anhand der klinischen Beurteilung der Beatmung und einer kontinuierlichen endtidalen CO_2-Detektion mit steigender Wellenform.
**Strategien zur erfolgreichen Extubation**	**Empfehlung der Leitlinie:**<br>• Verwenden Sie nicht-invasive Unterstützung der Atmung falls dies nach der Extubation notwendig ist (nasale High-Flow-Oxygenierung, kontinuierliche positive Atemwegsbeatmung oder nasale intermittierende Überdruckbeatmung).<br>**Praxis-Tipp der Autoren:**<br>• Bei jeder Extubation muss die Ausrüstung für eine sofortige Reintubation vorhanden sein, wobei die spezifischen Besonderheiten des Kindes, der vorherigen Atemwegssicherung und die Ursachen einer vorherigen schwierigen Intubation bei dieser Vorbereitung berücksichtigt werden müssen.<br>• Verwenden Sie Kortikosteroide und/oder vernebeltes Epinephrin zur Vorbeugung und Behandlung von Stridor nach der Extubation, wenn die Atemwege erheblich manipuliert wurden.<br><br>**Schlafende oder wache Extubation**<br>**Praxis-Tipp:**<br>• Wenn die Maskenbeatmung und die Intubation problemlos waren sowie keine klinischen erhöhten Risiken für Atemwegskomplikationen (Infobox 1) vorliegen, sind eine altersgerechte Atemfrequenz mit Tidalvolumina von mindestens 5 ml/kg Kriterien für die Extubation.
**Human Factors und personelle Kompetenzen für das Atemwegsmanagement beim Säugling**	**Praxis-Tipp:**<br>• Eine gute Zusammenarbeit im Team, zielgerichtete Kommunikation sowie eine Atmosphäre mit einer gesunden Fehlerkultur können Patientenschäden reduzieren.<br>• Das Atemwegsmanagement bei Neugeborenen und Säuglingen erfordert eine Reihe spezifischer Fähigkeiten und ein strukturiertes Training. Es ist ratsam, die Fähigkeiten mit Hilfe eines Simulationstrainings zur neonatalen und Säuglings-Intubation zu implementieren und aufrechtzuerhalten.
